# Water Availability and Temperature as Modifiers of Evaporative Water Loss in Tropical Frogs

**DOI:** 10.1093/icb/icae057

**Published:** 2024-06-05

**Authors:** Bryan H Juarez, Isaac Quintanilla-Salinas, Madison P Lacey, Lauren A O'Connell

**Affiliations:** Department of Biology, Stanford University, Stanford, CA 94305, USA; Earth System Science Department, Stanford University, Stanford, CA 94305, USA; Mathematics Department, California State University Channel Islands, Camarillo, CA 93012, USA; Department of Biology, Stanford University, Stanford, CA 94305, USA; Department of Biology, Stanford University, Stanford, CA 94305, USA

## Abstract

Water plays a notable role in the ecology of most terrestrial organisms due to the risks associated with water loss. Specifically, water loss in terrestrial animals happens through evaporation across respiratory tissues or the epidermis. Amphibians are ideal systems for studying how abiotic factors impact water loss since their bodies often respond quickly to environmental changes. While the effect of temperature on water loss is well known across many taxa, we are still learning how temperature in combination with humidity or water availability affects water loss. Here, we tested how standing water sources (availability) and temperature (26 and 36°C) together affect water loss in anuran amphibians using a Bayesian framework. We also present a conceptual model for considering how water availability and temperature may interact, resulting in body mass changes. After accounting for phylogenetic and time autocorrelation, we determined how different variables (water loss and uptake rates, temperature, and body size) affect body mass in three species of tropical frogs (*Rhinella marina, Phyllobates terribilis*, and *Xenopus tropicalis*). We found that all variables impacted body mass changes, with greater similarities between *P. terribilis* and *X. tropicalis*, but temperature only showed a notable effect in *P. terribilis*. Furthermore, we describe how the behavior of *P. terribilis* might affect its water budget. This study shows how organisms might manage water budgets across different environments and is important for developing models of evaporative water loss and species distributions.

## Introduction

Hydro- and thermoregulation are critical elements of physiological processes in all organisms (Potts 1994; Chown et al. 2011; Lombardini and Rossi 2019; Rozen-Rechels et al. 2019). Water is necessary for the transport and maintenance of solute concentrations found in the body, which may be altered by temperature and water loss (Takei and Hwang 2016). The importance of temperature in organisms ranges from controlling the rates of chemical reactions to the rates of organism-level traits, such as metabolic rates (Robinson et al. 1983; Hochachka and Somero 2002; Clarke and Fraser 2004; Angilletta 2009). While water and temperature are distinct aspects of the abiotic environment, their effects may be correlated (Riddell et al. 2019), and organisms often have to respond to changes in water and temperature in tandem. Terrestrial organisms that are challenged by relatively hotter and drier conditions must hydro- and thermoregulate or risk experiencing lethal levels of dehydration or heat stress (Kearney et al. 2013; Dupoué et al. 2017; O'Sullivan et al. 2017; MacMillan 2019). Mortality caused by dehydration or heat stress can have important ecological effects, including changes to population sizes or species distributions (Kearney et al. 2018; Camacho et al. 2023). For example, species distribution models that account for the effects of water loss on the voluntary thermal limits of lizards outperform those that do not (Camacho et al. 2023). However, relative to temperature, we have a limited understanding of the role of water loss in acclimation to hydrothermal environments (Weaver et al. 2023), shifting geographical distributions (Kearney et al. 2018), and vulnerability to climate change (Pintor et al. 2016; Riddell et al. 2023). Thus, learning how organisms are impacted by water loss and temperature will help us support conservation efforts for at-risk species.

Measurements of evaporative water loss (EWL) rates are commonly used to understand how organisms in terrestrial environments respond to hydrothermal challenges. Most EWL happens through fluid exchange along moist surfaces in the respiratory, sensory, and integumentary systems like the lungs, eyes, and skin (Mautz 1980; Senzano and Andrade 2018; Pirtle et al. 2019). EWL is determined by various factors, including water vapor pressure, temperature (Riddell et al. 2019), and traits such as body size, skin resistance, or metabolic rate (Eynan and Dmi'el 1993; Oufiero and Van Sant 2018; Senzano and Andrade 2018; Howard et al. 2020). EWL rates are important for understanding how organisms manage their water budgets, or water influx and efflux, across different environments (Kearney et al. 2013; Gouveia et al. 2019; Pirtle et al. 2019). Quantifying water loss is key for learning how organisms respond to changes in mass and about the resulting correlations between mass and traits such as body temperature, metabolism, and movement ability (Losos 1990; Marsh 1994; Iriarte-Díaz 2002).

There are two major challenges to learning how water loss may impact organismal traits across different environments. The first challenge is determining whether water and temperature have non-additive effects on EWL and body mass (Kearney et al. 2013; Rozen-Rechels et al. 2019; Weaver et al. 2023). For instance, water may reduce EWL by covering the body and limiting body surface area exposed to air. Furthermore, some organisms might offset EWL by absorbing water from the environment (Dainton 1954; Withers 1993; Yoder et al. 2007). A second challenge is determining how organisms behaviorally modify how they experience their abiotic environment to alter dehydration rates (Pough et al. 1983; Pirtle et al. 2019; Dezetter et al. 2023). For example, many animals burrow to avoid dehydration or use evaporative cooling to reduce their body temperature (Hall and Root 1930; Duellman and Trueb 1994). Learning how water and temperature contribute to dehydration in the context of water sources and behavior is necessary to understand how organisms perform hydro- and thermoregulation.

Anuran amphibians are an ideal system for determining relationships between water loss, water absorption, temperature, and body size. Frogs have a rich 200+ year history of research describing how they manage their water budgets (Jørgensen 1997). While endotherms exhibit body temperatures above ambient temperatures, ectotherms maintain temperatures at or near ambient levels, relative to endotherms (Withers 1992; Muñoz-Garcia et al. 2014). This makes frogs useful in quantifying the effect of temperature on water loss. Furthermore, anurans exhibit a variety of integumentary, circulatory, and behavioral adaptations for delaying or slowing dehydration (Toledo and Jared 1993; Lemenager et al. 2022). Some examples include lipid secretions, integumentary cocoons, increased skin texturing, or water conservation postures (Jørgensen 1997). Notably, frogs use vascularization along the pelvic skin (the pelvic patch) to absorb water instead of drinking (Parsons and Mobin 1991; Suzuki et al. 2007). Frogs may also manage their water budget through behavior by climbing, jumping, or burrowing (Duellman and Trueb 1994) to reach new microhabitats that provide water or shelter from drying conditions (Brusch et al. 2019; Gastón and Akmentins 2023). Additionally, traits used in managing water budgets (Toledo and Jared 1993), including behavioral and phenotypic traits, are often linked to microhabitat use. For example, terrestrial frogs exhibit some of the lowest rates of dehydration among frogs while aquatic frogs exhibit some of the highest (Young et al. 2005; Tracy et al. 2014; Cruz-Piedrahita et al. 2018). Learning how frogs manage their water budgets should help us develop better predictions of how ectothermic organisms respond to extreme environments and climate change.

While we generally understand water budgets in temperate frogs, we have a much narrower understanding of water budgets in tropical frogs (Scheffers et al. 2013; Reider et al. 2021; Womack et al. 2022; Bovo et al. 2023). This is a critical knowledge gap because most of the world’s frogs (>7000 species) are found in the tropics (Jenkins et al. 2013). Globally, 50% of frogs are classified as threatened, endangered, or critically endangered, and the world may face catastrophic losses of amphibian biodiversity in the tropics without intervention (Stuart et al. 2004; González-Del-Pliego et al. 2019). In this study, we chose to learn about water loss in three species: cane toads (*Rhinella marina*), golden poison frogs (*Phyllobates terribilis*), and Western clawed frogs (*Xenopus tropicalis*). We chose these species by selecting primarily terrestrial or aquatic frogs with large expected differences in EWL. *Rhinella marina* is a large (∼85–225 mm; AmphibiaWeb 2023) primarily terrestrial toad native to Central and South America. *Rhinella marina* is also a prevalent invasive species around the world and has high skin resistance to dehydration (Kosmala et al. 2020). *Phyllobates terribilis* is a small (∼46 mm; AmphibiaWeb 2021) terrestrial frog native to Colombia. To our knowledge, dehydration rates have not been measured in *P. terribilis* but we expect it to experience rapid dehydration due to its small size (Tracy et al. 2010). *Xenopus tropicalis* is a small (∼28–40 mm; AmphibiaWeb 2024) aquatic frog native to Western and Central Africa with low skin resistance to dehydration (Mokhatla et al. 2019).

**Fig. 1:**
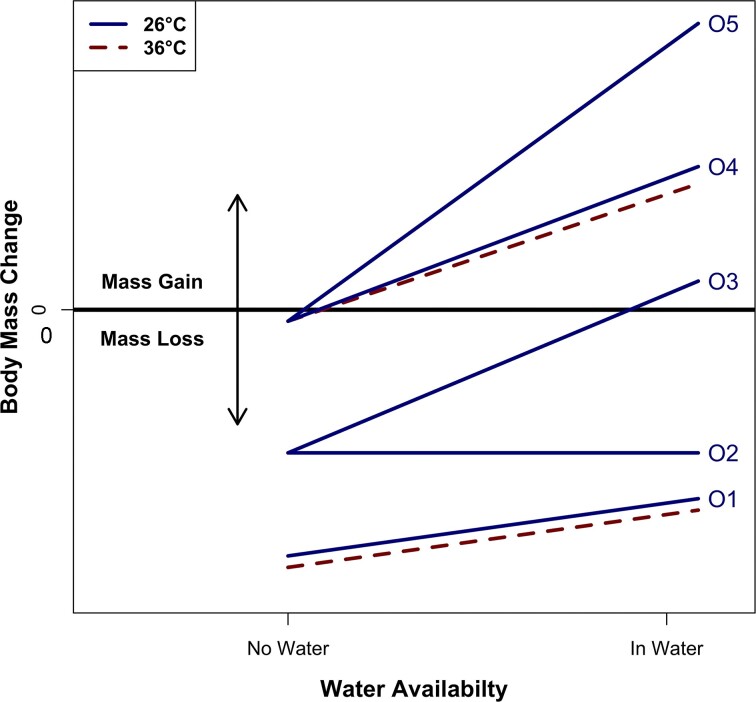
A conceptual model of how body mass may change due to interactions between water availability and temperature. These examples are not exhaustive. We assume mass gain is only possible in water and relatively higher temperatures always result in greater mass loss. Outcomes (O) 1–5 show treatment effects (solid blue lines) for water at 26°C. For simplicity, we only show two 36°C (dashed red) lines near the lower slope = 0 limit for each potential outcome, corresponding to the lack of an interaction with temperature for O1 and O4. 36°C treatments may also yield results as shown in O1–5, representing an interaction (if statistically distinct from the 26°C effect). O1 and O4 show no temperature interaction and only a small effect of water availability, resulting in mass gain or mass loss. O2 shows no marginal effect of water. O3 and O5 show stronger possible temperature interactions (relative to 36°C). Relative to O5, O3 shows more body mass is lost without water, and only a small body mass increase when in water.

The goal of this study is to determine how frogs respond to the dual effects of water availability and temperature. We use *R. marina, P. terribilis*, and *X. tropicalis* to test the hypothesis that water can buffer against EWL by limiting body surface area exposed to air. Alternatively, water sources may not affect EWL if the animals do not use water sources, as this would not alter the body surface area exposed to air. We demonstrate in Fig. 1 how water availability may buffer or overcome EWL due to temperature, resulting in mass loss or mass gain. Whether mass is lost or gained in each environment depends on the relative rates of water uptake and evaporation. Additionally, we hypothesize that larger bodies are more resistant to EWL since larger organisms have a relatively lower surface area-to-volume ratio. Lower ratios limit the surface area over which evaporation might happen, relative to volume (Gouveia et al. 2019; Castro et al. 2021). We also expect that the extent to which hydrothermal environments affect body mass varies across species. We predict that *R. marina* is the most resistant to EWL, followed by *P. terribilis*, and then *X. tropicalis* based on our expectation that terrestrial frogs should experience less EWL than aquatic frogs. Overall, this study advances our understanding of ecology in ectotherms by determining how water budgets are managed in different environments and is important for developing our models of EWL and species distributions.

## Methods

### Specimen collection and housing

Wild *R. marina* (*N* = 32) were obtained from Oahu, Hawaii, USA (HI Permit No. EX-23-04). We caught these toads by hand and placed them in containers with moist soil and a water source prior to and during shipping to Stanford, CA. *Phyllobates terribilis* (*N* = 31) were purchased from Indoor Ecosystems (Whitehouse, Ohio, USA). *Xenopus tropicalis* (*N* = 32) were purchased from Xenopus 1, Corp. (Dexter, Michigan, USA). All animal procedures were conducted in compliance with Stanford University’s research ethics review board (APLAC 34069).

Each animal was placed in species-specific housing. These tanks were approximately 101.0 cm L x 54.6 cm W x 45.5 cm H for the *R. marina* and 76.2 cm L × 45.7 cm W × 45.7 cm H for the other species. *Rhinella marina* and *P. terribilis* were housed socially in plastic tanks in photoperiod-, temperature-, and humidity-controlled rooms with a 12 h light-dark cycle (night from 3 pm to 3 am) at an average of 26°C and 100% humidity. The animal tanks contained moist soil, sphagnum moss, and water sources for each species (e.g., water in petri dishes or glass bowls). We also included shelter for each species consisting of *Philodendron* plants and/or coconut endocarp. *Rhinella marina* and *P. terribilis* were misted daily and fed every other day. We provided each toad three crickets and each poison frog roughly 60 flies on each feeding day. We provided approximately 60 flies per frog to make up for the fact that a large portion of flies escape or are not eaten, and therefore the real number of flies eaten is likely far less. *Xenopus tropicalis* were housed socially in aquaria at 28°C and a 12 h (night from 9 pm to 9 am) photoperiod. The aquaria included shelters made of PVC. *Xenopus tropicalis* tanks received a daily 20% water change, and each frog was fed approximately five aquatic frog pellets every other day. Animals were kept in these standard housing conditions for at least one week before data collection.

### Data collection

We exposed each frog to a starvation period of at least 3 days prior to acclimation and application of treatments and did not feed any animals after the first day of experimentation. This procedure let us standardize for potential metabolic effects on body mass, independent of body size differences between species. We assigned frogs randomly to experimental treatments and exposed them to an acclimation period of 24 h prior to applying each treatment. Following the starvation period, we transferred each frog to individual plastic terraria for 24 h (at 26°C) to acclimate them to their experimental containers. We used larger containers (29.8 cm L × 19.7 cm W × 20.3 cm H) for *R. marina* and smaller containers (23.5 cm L × 15.2 cm W × 17.8 cm H) for the other species. To prevent stress and mortality during this period, we provided the frogs with shelter and a water source (*R. marina* and *P. terribilis*) or enough water to cover their bodies and the shelter (*X. tropicalis*). We removed the shelter and water sources and dried the individual tanks with a paper towel, as needed, immediately prior to applying each water and temperature treatment. We measured four frogs per day (one per treatment) for 8 days (4 frogs/day × 8 days = 32 frogs).

We exposed each species to four treatments, including all combinations of water presence/absence and incubation at 26 or 36°C, resulting in an average of *N* = 8 samples per treatment (32 frogs/4 treatments) for each species. Following Shibata et al. (2014), we filled each container with 1 cm of water for the water presence treatment and provided a 1 cm L × 1 cm W × 0.5 cm H moist sponge to frogs in the water absence treatment to minimize potential discomfort but limit potential water absorption. We used a Fisherbrand™ Isotemp™ BOD Refrigerated Incubator to control experimental temperatures. We weighed each frog prior to each treatment and approximately every 20 min for an hour, since preliminary experiments indicated high mortality for *P. terribilis* for longer periods. We gently patted each frog with a paper towel to remove excess water prior to weighing. We euthanized each frog by administering intracoelomic injections of 1% MS-222 followed by decapitation at the conclusion of the experiment after 1 h, or after individuals: (1) lost > 20% body mass, or (2) did not exhibit a righting response. We recorded body size (snout-vent length) for each frog immediately after administering MS-222 but before decapitation.

### Data analysis

All analyses were done in R 4.3.2 (R Core Team 2024). We implemented some corrections to account for urination, defecation, and missing values. In total, only six *R. marina* and two *P. terribilis* urinated and/or defecated (0.5–3% body mass) throughout the experiment. Since mass changes associated with urination and defecation are not due to evaporation, we corrected the raw body masses by adding the weight of the urine and stool to prevent overestimation of the EWL. Furthermore, we removed *N* = 9 individuals from the study due to lack of righting response or >20% mass loss. Removing the *N* = 9 individuals resulted in ten total missing values for body mass at various times. We estimated these missing values by predicting body mass at each corresponding time point using a linear regression of body mass and time with all available data. Lastly, we could not measure the mass of an additional *N* = 8 frogs for one time point per frog since the measurement period overlapped with endpoints for other frogs. In these cases, we estimated the missing values as the average mass of the previous and next measurements. For the whole study, we had *N* = 18 missing mass values of a possible 380 measurements (4.74%).

We analyzed the data in a Bayesian framework by fitting a phylogenetic longitudinal generalized linear mixed-effects model using a Hamiltonian Monte Carlo algorithm implemented using Stan 2.26.1 (Stan Development Team 2024), which we accessed using the R library cmdstanr (Gabry et al. 2024). We used four Markov Chains with each chain having 30,000 burn-in and 50,000 samples. We verified appropriate model fit using standard diagnostics for multi-level longitudinal models (Fitzmaurice et al. 2004). We regressed body mass (natural log grams) onto time (minutes), water availability, temperature, and snout-vent length (natural log mm). We included interactions between water and temperature to test our hypothesis that EWL depends on water availability and a second interaction between water and time to model separately the time effects associated with water loss and water uptake. We also included random effects for individuals, housing group, date, and species. To account for phylogenetic and time autocorrelation, we used a composite covariance matrix obtained from the error variance and correlation matrix. We obtained the phylogenetic correlation matrix after estimating mean branch lengths from 1000 trees drawn from the pseudo-posterior distribution of Jetz and Pyron (2018). Since we did not measure body mass at exact 20 min intervals, we modeled temporal autocorrelation using a Gaussian function that incorporates the time difference between measurements (Diggle et al. 2013).

We modeled all parameters of interest and the corresponding priors using normal or inverse gamma distributions, where appropriate (Gelman et al. 1995). We designed priors with mean effects estimated from preliminary experiments on *P. terribilis* or prior literature (Mokhatla et al. 2019; Kosmala et al. 2020) and enough variance for the effects to include zero on the raw scale. We allowed water uptake rates to be up to double the dehydration rates based on prior evidence that water uptake can happen very quickly (Jørgensen 1997). The prior for the water-temperature interaction assumed that on average, half the surface area of *X. tropicalis* and one-quarter of the surface area of *P. terribilis* and *R. marina* were covered with water in those treatments (based on our observations). The priors for body size were set at the midpoints of the minimum and maximum body sizes for each species. The variance priors were modeled with an inverse-gamma distribution with error variance modeled with a narrow range and the random effects variances modeled with a wide range. The correlation parameter was modeled as uniform distribution. We list and describe all prior distributions in the Supplementary Materials (Table S1).

## Results

**Fig. 2:**
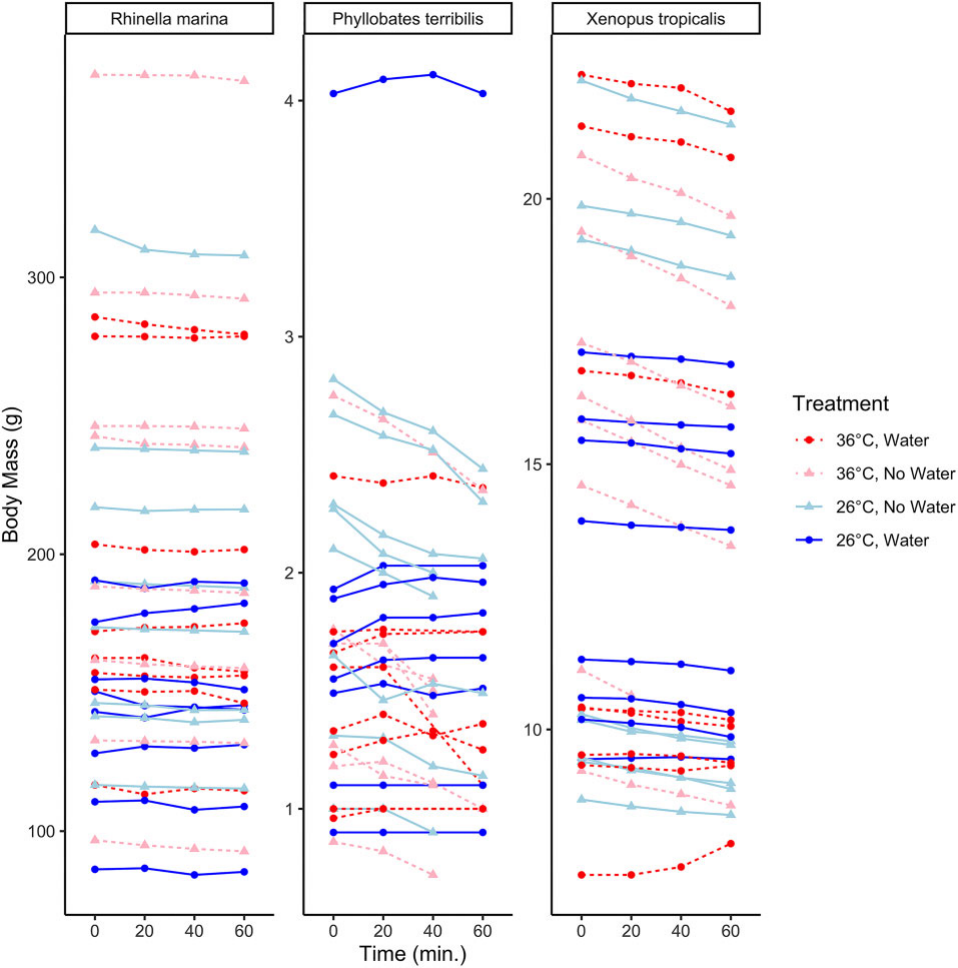
Raw body mass data were collected over 1 h for each water availability and temperature treatment. Each panel is a different species. Red hues correspond to 36°C, and blue hues correspond to 26°C. Circles show water presence and triangles show water absence treatments. We assumed even sampling at 20 min intervals for plotting purposes, but we analyzed the real times at which measurements were taken (see the “Methods” section).

**Fig. 3:**
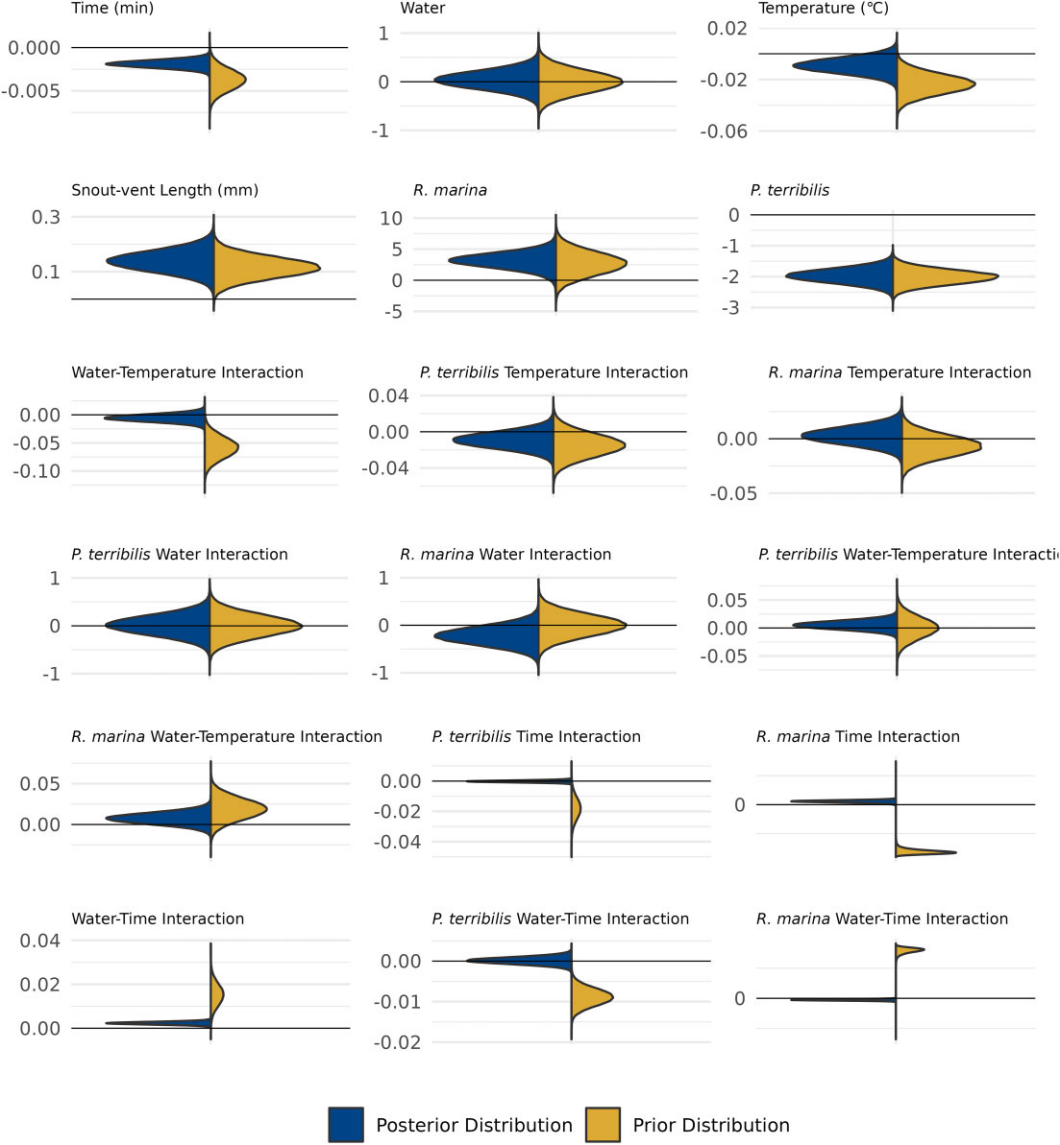
Prior and posterior distributions of model effects. Posterior distributions (left) are shown in blue, and prior distributions (right) are shown in yellow. Snout-vent length is a measure of body size in amphibians. All prior distributions shown here were normally distributed. Please see text for how we chose to specify the mean and variance for each prior.

Generally, our raw data show that most instances of water loss occur in the treatments without water (Fig. 2). We also plotted individual stepwise changes in mass through time in the Supplementary Materials (Fig. S1). Comparison of the posterior and prior distributions of each model effect shows how our data updated our prior expectations, generally resulting in better estimates of mean effects with higher precision (Fig. 3). Summary plots for evaluating model fit are found in the Supplementary Materials.

We found larger body sizes and water availability reduce EWL and these effects, like the EWL rate, differ between species (Table 1). For instance, we found larger bodies prevented dehydration at an average rate of 14.83% body mass per 2.72 mm increase in snout-vent length (=0.1383 log g/log mm) after 30 min in *Xenopus*. We found water availability reduces net EWL by allowing for water uptake. This effect was similar for *X. tropicalis* and *P. terribilis* at an average rate of 0.23% body mass per minute (=0.0023 log g/min), whereas *R. marina* did not absorb any water. We also found *X. tropicalis* and *P. terribilis* showed similar EWL rates of 0.2% body mass per minute (=0.0019 log g/min), whereas *R. marina* lost less mass over time at a rate of 0.02% body mass per minute (=0.0002 log g/min). Each of these effects had 95% credible intervals that excluded 0. Furthermore, we found frog ID, housing, and experimental date did not explain much variation in body masses, especially compared to interspecific differences. We found interspecific differences accounted for 20, 40, and 65 times more variance than housing, date, and frog ID, respectively.

**Table 1:** Summary of posterior distributions of factors affecting dehydration

Model term	Mean	Q2.5	Q97.5
Intercept (ln g)	0.0887	−0.3665	0.5457
**Time (ln g/min, not in water)**	**−0.0019**	**−0.0028**	**−0.0010**
Water (ln g, not in water)	0.0373	−0.3286	0.4024
Temp. (ln g/°C, not in water)	−0.0092	−0.0208	0.0023
**SVL (ln g/ln mm)**	**0.1383**	**0.0633**	**0.2137**
***R. marina***	**3.1515**	**0.5209**	**5.6464**
***P. terribilis***	**−1.9741**	**−2.4284**	**−1.5218**
*R. marina*-Temp.	−0.0057	−0.0186	0.0070
*P. terribilis*-Temp.	−0.0092	−0.0272	0.0086
Water-Temp. (ln g/°C, in water)	0.0028	−0.0130	0.0186
*P. terribilis*-Water	0.0139	−0.3864	0.4134
*R. marina*-Water	−0.2219	−0.6034	0.1616
*P. terribilis*-Water-Temp.	0.0051	−0.0098	0.0201
*R. marina*-Water-Temp.	0.0074	−0.0063	0.0211
*P. terribilis*-Time	−0.0002	−0.0014	0.0011
***R. marina*-Time**	**0.0017**	**0.0005**	**0.0030**
**Water-Time (ln g/min, in water)**	**0.0023**	**0.0011**	**0.0035**
*P. terribilis*-Water-Time	0.0001	−0.0016	0.0018
***R. marina*-Water-Time**	**−0.0023**	**−0.0040**	**−0.0005**
Frog ID variance	0.0787	0.0535	0.1146
Housing variance	0.2555	0.1163	0.5413
Date variance	0.1280	0.0688	0.2340
Species variance	5.1479	0.9350	19.6249
Error variance	0.0174	0.0135	0.0224

We obtained these results using a phylogenetic time series generalized linear mixed model implemented in a Bayesian framework. Mean is the mean of the effect. Q2.5 and Q97.5 are the quantiles corresponding to the two-tailed 95% credible interval. Rows corresponding to main effects in bold exclude 0 from the 95% credible interval. SVL is snout-vent length. *R*. is *Rhinella. P*. is *Phyllobates*. Housing is a grouping factor indicating the experimental housing. Date is the date of measurement. We provide units for each effect on the baseline of *X. tropicalis*. ln is natural log. Temp. is temperature.

**Fig. 4:**
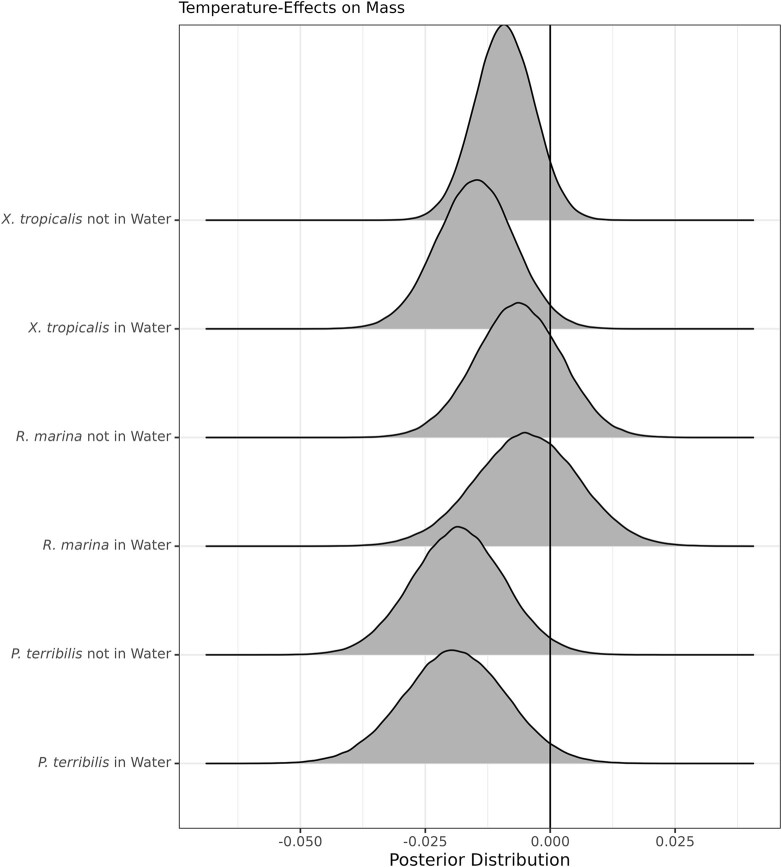
Posterior distributions of the marginal effect of temperature by species and water treatment. These effects correspond to body mass. The height of each distribution corresponds to the probability density. The vertical line denotes an effect of 0.

While temperature seemed unimportant at first, we found higher temperatures increased EWL in *X. tropicalis* and *P. terribilis*. We estimated the posterior marginal effects of temperature for each species across water treatments and plotted them (Table 2; Fig. 4). We found *P. terribilis* experienced greater water loss at higher temperatures (when not in water) at a rate of 1.02 g/°C (0.0185 log g/°C). The 95% credible interval for the previous result excluded 0. Interestingly, the marginal effect of temperature for *P. terribilis* in water is visually similar to that of *P. terribilis* not in water (with similar quantiles and mean). While the 95% credible intervals corresponding to *P. terribilis* in water and *X. tropicalis* in both water treatments included 0, we also found very high probabilities (91.14–97.32%) that higher temperatures contribute to greater dehydration in *X. tropicalis* and *P. terribilis*, regardless of water treatment.

**Table 2:** Summary of the posterior distributions of the marginal effects of temperature across species and water treatments

Model term (ln g/°C)	Mean	Q2.5	Q97.5
*R. marina* in water	−0.0047	−0.0252	0.0153
*R. marina* not in water	−0.0064	−0.0235	0.0104
*P. terribilis* in water	−0.0191	−0.0393	0.0010
***P. terribilis* not in water**	**−0.0185**	**−0.0365**	**−0.0004**
*X. tropicalis* in water	−0.0150	−0.0304	0.0002
*X. tropicalis* not in water	−0.0092	−0.0208	0.0023

Mean is the mean of the effect. Q2.5 and Q97.5 are the quantiles for the two-tailed 95% credible interval. Rows corresponding to effects in bold exclude 0 from the 95% credible interval. ln is the natural log.

## Discussion

To our knowledge, this is one of the first studies to examine the dual effects of water sources and temperature on EWL. Overall, we found body masses change in predictable ways due to EWL, water uptake, size, and temperature. While we did not find an interaction between water and temperature, we did find interspecific differences in how temperature affected EWL. Our results support our hypotheses that water sources dampen rates of EWL and that the precise mechanisms affecting EWL vary by species. Contrary to our expectation, rates of water uptake and EWL were most similar between the aquatic *X. tropicalis* and terrestrial *P. terribilis*, rather than between both species with terrestrial lifestyles. The latter result supports assertions that species-specific traits may control water budgets (e.g., Pirtle et al. 2019), but further research is needed to determine how body size and ecology are related to water uptake.

Compared to prior research, we found differences in the rates of water loss and uptake in *R. marina*, but these may be explained by differences in body size, temperature, or experimental design. We found the *R. marina* experienced lower rates of EWL than *X. tropicalis* and *P. terribilis* at ∼33 mg/min, corresponding to a median starting mass of 165 g and mean experimental temperature of 31°C. This rate is almost four times previous estimates of 8.8 mg/min at 26°C (Kosmala et al. 2020) for frogs weighing 110 g. Since larger bodies and hotter temperatures are associated with higher rates of evaporation (Mautz 1980), much of the difference can be explained by body mass and temperature alone. Kosmala et al. (2020) also report a water uptake rate of 0.5 g/min, whereas *R. marina* in this study did not absorb any appreciable amount of water. This major difference is likely due to Kosmala et al. (2020) measuring water uptake in toads that had lost 30% body mass, whereas we allowed our toads to hydroregulate at will prior to experimentation. Based on our findings for *R. marina*, it is evident that spending 1 h in drying conditions (with or without water) had little impact on this species’ body mass (Fig. 2). Since *R. marina* did not absorb much water even after spending 1 h at 36°C, this suggests these conditions were either not stressful to this species or did not last long enough to provoke the frogs to absorb water. This result shows how understanding responses of frogs to specific environmental conditions might benefit from further research on hormones like arginine vasotocin, which is linked to dehydration and rehydration in frogs (Morel and Jard 1963; Cartledge et al. 2008; Uchiyama et al. 2014).

We also found differences in our estimated rates of EWL and those in other studies for *X. tropicalis*. We found *X. tropicalis* (average body mass = ∼13.5 g) dehydrated at a rate of 12% body mass/h, which is higher than previous findings of 1.6% body mass/h (Mokhatla et al. 2019) in *Xenopus laevis* (average body mass = ∼57 g). Since larger frogs dehydrate at a slower rate, body mass alone explains much of this difference, but the reported rate of 1.6% mass/h seems to be an average across temperatures ranging from 0 to 35°C, complicating direct comparisons. Furthermore, our estimates of water uptake (∼0.0322 g/min) were similar to previous findings in anesthetized *X. laevis* who were roughly twice as large as our animals and whose rate of water uptake was 0.012 g/min (Ireland 1973), given animals with a mean body mass of 27.5 g. The latter study did not report an experimental temperature. Comparing rates of water transfer depends on standardizing a variety of variables and while we explain how body size, temperature, and experimental design might account for our observed differences, humidity might be an additional confounding factor that should be investigated in future research.

While *P. terribilis* and *R. marina* share a terrestrial lifestyle, *P. terribilis* seems to manage its water budget more similarly to the aquatic *X. tropicalis*. In other words, while shared ecology is sometimes informative of EWL (Wygoda 1984), it may not always be a reliable indicator of EWL. Additionally, unlike the other two species, there is little literature on how *P. terribilis* manages its water budget. While *P. terribilis* and *X. tropicalis* had similar water loss and uptake rates, *P. terribilis* showed greater dehydration at higher temperatures when not in water. Since they had similar water loss and uptake rates, this means the two species differ in some other factor related to rates of water transfer. We suspect *P. terribilis* has a higher skin resistance relative to *X. tropicalis*, making water transfer rates similar despite temperature having a greater impact on *P. terribilis*. To our knowledge, however, no studies have measured skin resistance in *P. terribilis*. We also showed how the marginal effects of temperature on *P. terribilis* were quite similar across water treatments. Anecdotally, we believe this is due to ∼25–50% of individuals refusing to remain in the water during the experiment and choosing to climb up to the corner of the tank instead. There might be two reasons why we observed this behavior. First, climbing might be a stress response where the frogs chose to seek shelter at the expense of water loss. Second, it is possible the animal was not stressed but chose to use the corner as a refuge to reduce the surface area directly exposed to the air, resulting in less EWL. Either way, this climbing behavior and the roles of stress hormones in hydro- and thermoregulation of poison frogs, or other frogs that can climb, are important areas of future study.

This study shows the importance of water sources when considering how EWL occurs in nature. Humidity (van Dyk et al. 2019) and hydration states (Senzano and Andrade 2018) are undoubtedly important factors that can interact with temperature to affect EWL. While we did not observe an interaction between water availability and temperature, this might be due to the short time and temperature range we used. For comparison, Senzano and Andrade (2018) ran their experiment for 2 h and over a 20°C range. Future work might show that water availability indeed interacts with temperature, since our prediction was founded on the theoretical premise that EWL rates depend on the animal’s surface area (Gouveia et al. 2019). In nature, water may change the exposed body surface area on frogs while they are in water or floating on it, after they rub against wet substrate (e.g., wet plants), or as it is raining. Two of the latter examples involve behaviors whose role in EWL is understudied (Tracy et al. 2014). As we might have observed with *P. terribilis*, organisms may seek hydric refugia (Kearney et al. 2013) and the types of available refugia depend on the environment. Previous studies have found that *R. marina* depends on water availability and not heat tolerance for moving across dry environments, although high heat tolerance enabled *Rhinella granulosa* to retain high jumping performance even at high temperatures (Prates et al. 2013; Brusch et al. 2019). Thus, we may generally expect that behaviors (like water conservation postures or jumping), together with the environment, determine how we should interpret the importance of dehydration (Davis and DeNardo 2010; Pintor et al. 2016; Pirtle et al. 2019; Dezetter et al. 2023).

Mitigating future amphibian declines depends on our ability to predict how different groups are affected by changing hydrothermal environments (Roznik et al. 2018; Greenberg and Palen 2021). Some have considered how acclimation to different environments might allow organisms to survive challenging conditions (Weaver et al. 2023). In this study, we exposed frogs to sharp environmental changes with no acclimation period. Evaluating how organisms respond to various environmental conditions, with and without acclimation, should allow us to determine which species might survive gradual versus extreme changes in climate, reflecting the spatial heterogeneity of climate change (Kaufmann et al. 2017). Furthermore, if climate change proceeds slowly in some regions, we may expect organisms to adapt. This implies an eco-evolutionary framework for thinking about global climate impacts on ectotherm diversity is needed. In the context of geographical ranges, water and temperature have unique effects on behavior and species distributions (Kearney et al. 2018; Delgado-Suazo and Burrowes 2022; Camacho et al. 2023). However, potential trade-offs between traits like water balance and jumping performance are also important for predicting how organisms will move throughout the landscape in response to climate change (Moore and Gatten 1989; Titon and Gomes 2015; Mitchell and Bergmann 2016). Since metabolic rates increase EWL by increasing respiratory rates (Preest et al. 1992; Tomlinson and Phillips 2012), considering abiotic effects on movement, behavior, and physiology seems crucial. In this context, we hope this study will be used to improve our models of EWL and ecogeographical gradients (Gouveia et al. 2019; Rubalcaba et al. 2019), and species distributions (Riddell et al. 2023). In summary, the future of amphibian conservation depends on understanding the complex relationships among many abiotic and biotic variables (Kearney et al. 2013; Schulte 2015; Gouveia et al. 2019; Rubalcaba et al. 2019). Future studies should seek to unravel how trade-offs or plastic responses of biotic variables, such as those associated with water balance or movement, are distributed both geographically and phylogenetically (Garland et al. 2022; Telemeco et al. 2022).

## Author contributions

B.H.J.: Conceptualization, Methodology, Software, Validation, Formal Analysis, Investigation, Resources, Data Curation, Writing—Original Draft, Writing—Review and Editing, Visualization, Project Administration, Funding Acquisition; I.Q.-S.: Methodology, Software, Validation, Formal Analysis, Resources, Data Curation, Writing—Review and Editing, Visualization; M.P.L.: Investigation, Resources, Writing—Review and Editing; L.A.O.: Conceptualization, Methodology, Resources, Writing—Review and Editing, Supervision, Project Administration, Funding Acquisition.

## Supplementary Material

icae057_Supplemental_File

## Data Availability

The data presented in this article are available in the Dryad Digital Repository (doi.org/10.5061/dryad.mw6m90650).

## Notes

From the symposium “Feel the flow: how water movement shapes organisms and ecosystems” presented at the annual meeting of the Society for Integrative and Comparative Biology, January 2–January 6, 2024.
