# Supplementary material for: Water Availability and Temperature as Modifiers of Evaporative Water Loss in Tropical Frogs
Source: Integr Comp Biol. 2024 Jun 5;64(2):354–65. doi: 10.1093/icb/icae057 (PMC11406161; doi:10.1093/icb/icae057)
Supplement: icae057_Supplemental_File [file icae057_supplemental_file.docx]

**Water availability and temperature as modifiers of evaporative water loss in tropical frogs**

**Authors:** Bryan H. Juarez^1,2,*^, Isaac Quintanilla-Salinas^3^, Madison P. Lacey^1^, and Lauren A. O’Connell^1^

^1^Department of Biology, Stanford University, Stanford, CA 94305, USA

^2^Earth System Science Department, Stanford University, Stanford, CA 94305, USA

^3^Mathematics Department, California State University Channel Islands, Camarillo, CA 93012, USA

*Corresponding author email: bryanhjuarez@gmail.com

**Supplementary Data**

We plotted stepwise changes in body mass for each individual and treatment (Fig. S1). The priors we used for each model term in our analyses are shown and described in Table S1. We found our Markov Chain Monte Carlo procedure for estimating model coefficients using a Bayesian framework showed good mixing properties using trace plots showing the range of samples taken for the log probability and model coefficients (Figs. S2–3). The QQ plot shows the model residuals fit a normal distribution well, with possibly more than expected probability density at the tails (Fig. S4). The residual plot shows no evidence of heteroscedasticity (Fig. S5). Lastly, we used a semivariogram to find very little evidence for residual autocorrelation (Fig. S6).


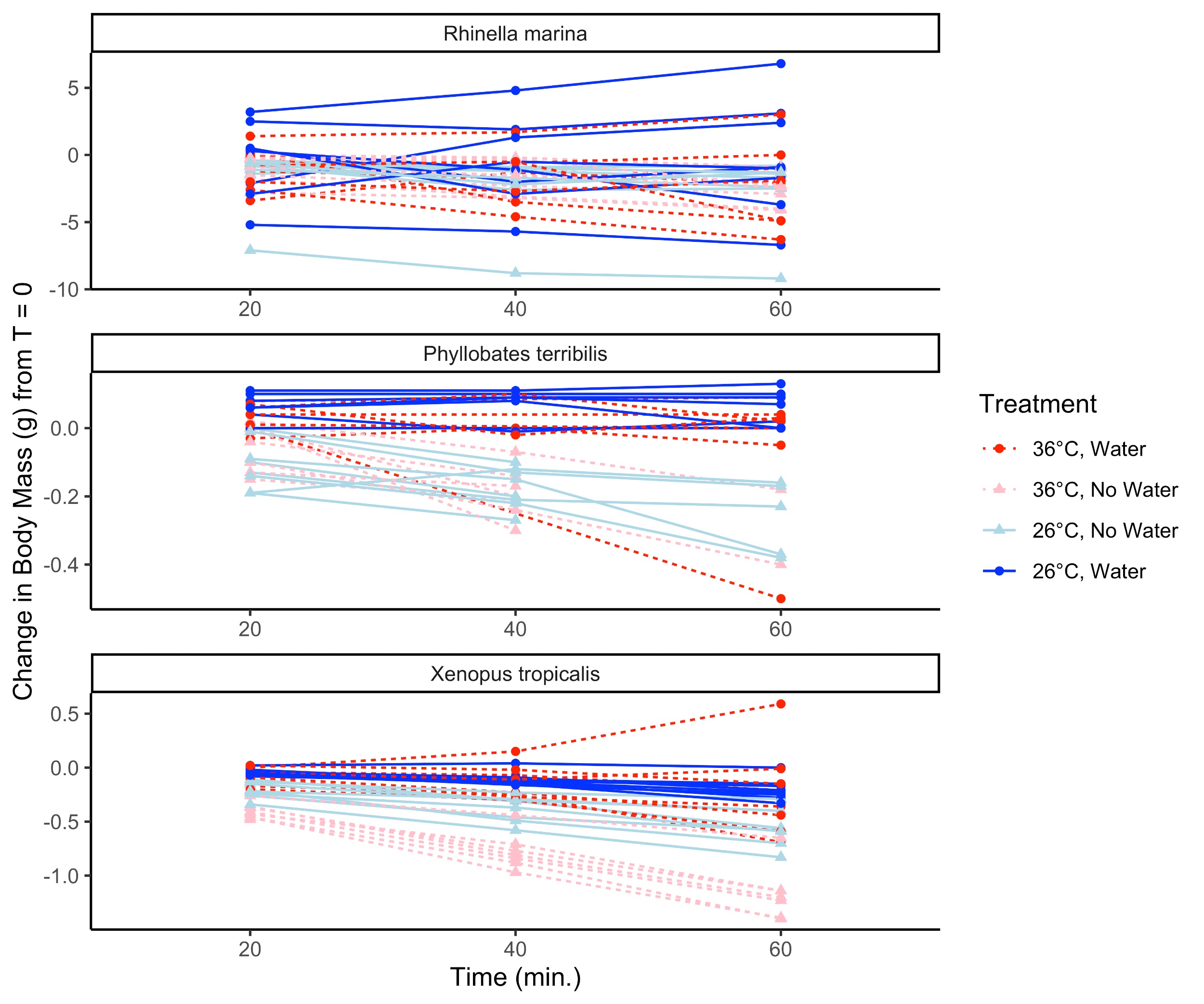


**Figure S1. Individual changes in body mass through time across water availability and temperature treatments.** The y-axis shows stepwise changes in time from the initial mass at Time = 0. The 36℃ treatments are shown using dashed lines (and red shades) while the 26℃ treatments are shown using solid lines (and blue shades). Treatments with water are shown in circles and those without water are shown in triangles.

**Alt text:** The plot shows differences through time in body mass, relative to each individual’s starting body mass. Each species has its own plot. The trends (regardless of treatment) for *Rhinella marina* are mostly flat. The trends for Phyllobates terribilis are more distinct between treatments with lack of water resulting in decreased body masses through time. Relative to the latter, the mass of *P. terribilis* in water at 26℃ does not change much through time. *Xenopus tropicalis* seems to decrease in mass through time, regardless of treatment.

**Table S1. Detailed description of priors used for different parameters in the Bayesian generalized linear mixed-effects model.** N is the normal distribution, IG is the inverse-gamma distribution and U is the uniform distribution. We list the parameters used for each in parentheses.

| **Term** | **Prior Distribution** | **Description** |
| --- | --- | --- |
| Intercept | N(0, 0.231) | The proposed log mass prior distribution for *X. tropicalis* at the beginning of the study not in the water treatment group. |
| Time | N(-0.004, 0.001) | The proposed change of log mass prior distribution for *X. tropicalis* per minute not in water. |
| Water | N(0, 0.231) | The proposed difference in log mass prior distribution for a *X. tropicalis* in water and out of water. |
| Temperature | N(-0.024, 0.008) | The proposed difference in log mass prior distribution for a *X. tropicalis* at 26℃ versus 36℃. |
| SVL | N(0.115, 0.038) | The proposed increase in log mass prior distribution for all species as SVL increases. |
| *R. marina* | N(2.686, 1.847) | The proposed difference in log mass prior distribution between *R. marina* and *X. tropicalis* not in the water treatment group. |
| *P. terribilis* | N(-2.015, 0.231) | The proposed difference in log mass prior distribution between *P. terribilis* and *X. tropicalis* not in the water treatment group. |
| Temperature-Water Interaction | N(-0.058, 0.019) | The proposed difference in log mass prior distribution for a *X. tropicalis* at 26℃ versus 36℃ in water. |
| *P. terribilis*-Temperature Interaction | N(-0.015, 0.013) | The proposed difference in log mass prior distribution for a *P. terribilis* at 26℃ versus 36℃. |
| *R. marina*-Temperature Interaction | N(-0.007, 0.01) | The proposed difference in log mass prior distribution for a *R. marina* at 26℃ versus 36℃. |
| *P. terribilis*-Water Interaction | N(0, 0.231) | The proposed difference in log mass prior distribution for a *P. terribilis* in water and out of water. |
| *R. marina*-Water Interaction | N(0, 0.231) | The proposed difference in log mass prior distribution for a *R. marina* in water and out of water. |
| *P. terribilis* Water-Temperature Interaction | N(0, 0.019) | The proposed difference log mass prior distribution between *P. terribilis* and *X. tropicalis* in water treatment group at 36℃. |
| *R. marina* Water-Temperature Interaction | N(0.019, 0.013) | The proposed difference log mass prior distribution between *R. marina* and *X. tropicalis* in water treatment group at 36℃. |
| *P. terribilis*-Time Interaction | N(-0.018, 0.007) | The proposed change of log mass prior distribution for *P. terribilis* per minute not in the water treatment group. |
| *R. marina*-Time Interaction | N(-4.394, 1.466) | The proposed change of log mass prior distribution for *R. marina* per minute not in the water treatment group. |
| Water-Time Interaction | N(0.015, 0.005) | The proposed change of log mass prior distribution for *X. tropicalis* per minute in water. |
| *P. terribilis* Time-Water Interaction | N(-0.009, 0.002) | The proposed difference in change of log mass prior distribution between *P. terribilis* and *X. tropicalis* per minute in water. |
| *R. marina* Time-Water Interaction | N(3.69, 1.235) | The proposed difference in change of log mass prior distribution between *R. marina* and *X. tropicalis* per minute in water. |
| Frog ID Random Effects Variance | IG(1,1) | Allow for large variances if larger random effects are needed. |
| Housing Random Effects Variance | IG(1,1) | Allow for large variances if larger random effects are needed. |
| Date Random Effects Variance | IG(1,1) | Allow for large variances if larger random effects are needed. |
| Species Random Effects Variance | IG(1,1) | Allow for large variances if larger random effects are needed. |
| Error Variance | IG(3,1) | Follows the Log Mass variability of the data. |
| Correlation parameter (𝜌) | U(0,1) | Allows the parameter to vary from 0 to 1 as defined in Diggle et al. 2013. |


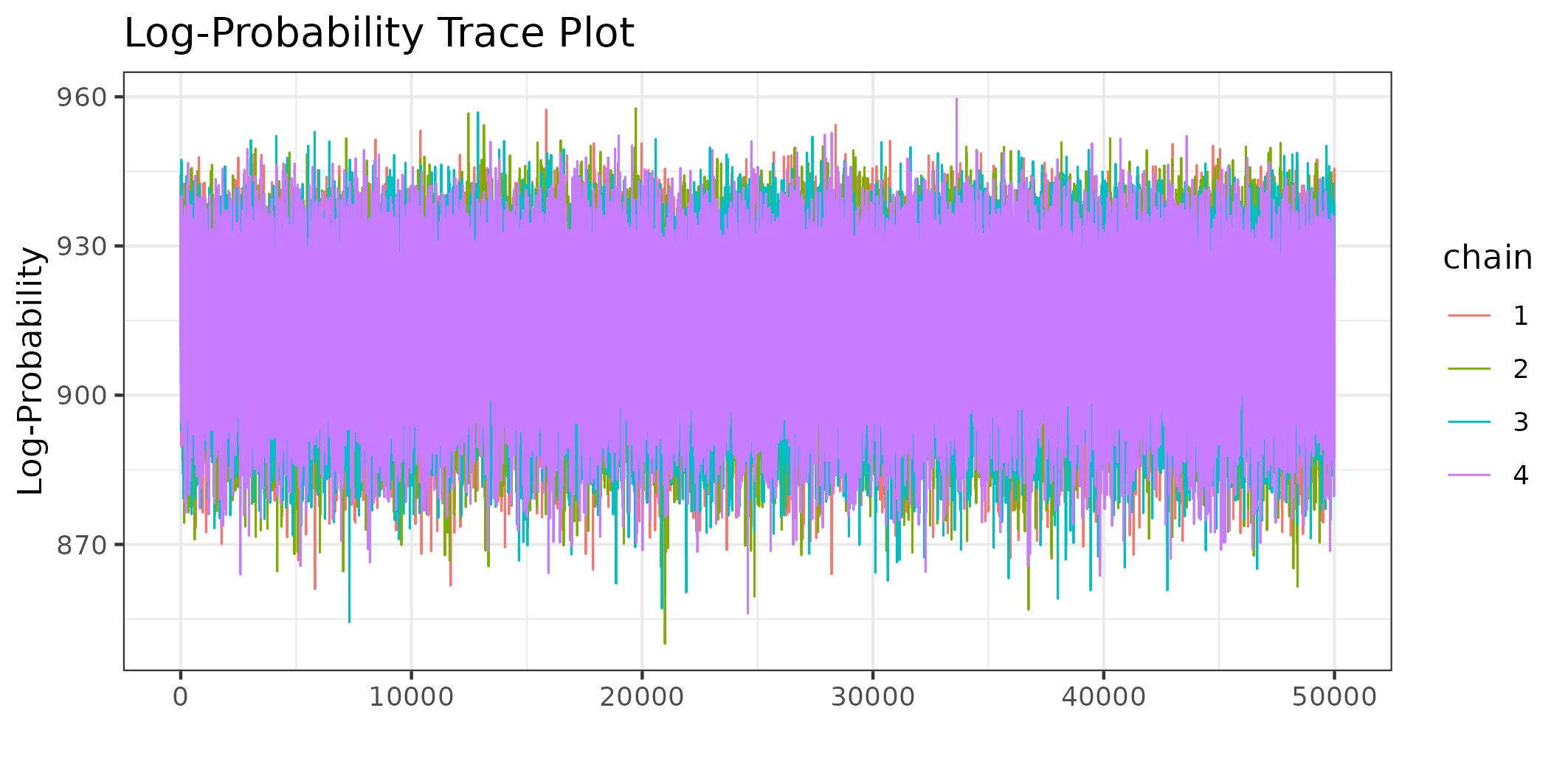


**Figure S2. Trace plot of the log-probability.** The x-axis shows each generation in the model up to 50,000 generations. We used 4 chains in the model, with each chain showing no trend and similar mixing properties with the sampled log-probabilites varying between about 855 and 960 across generations.

**Alt text:** Log-probability is on the y-axis and the model generation is on the x-axis. The data for each chain is similar with no trend across generations.


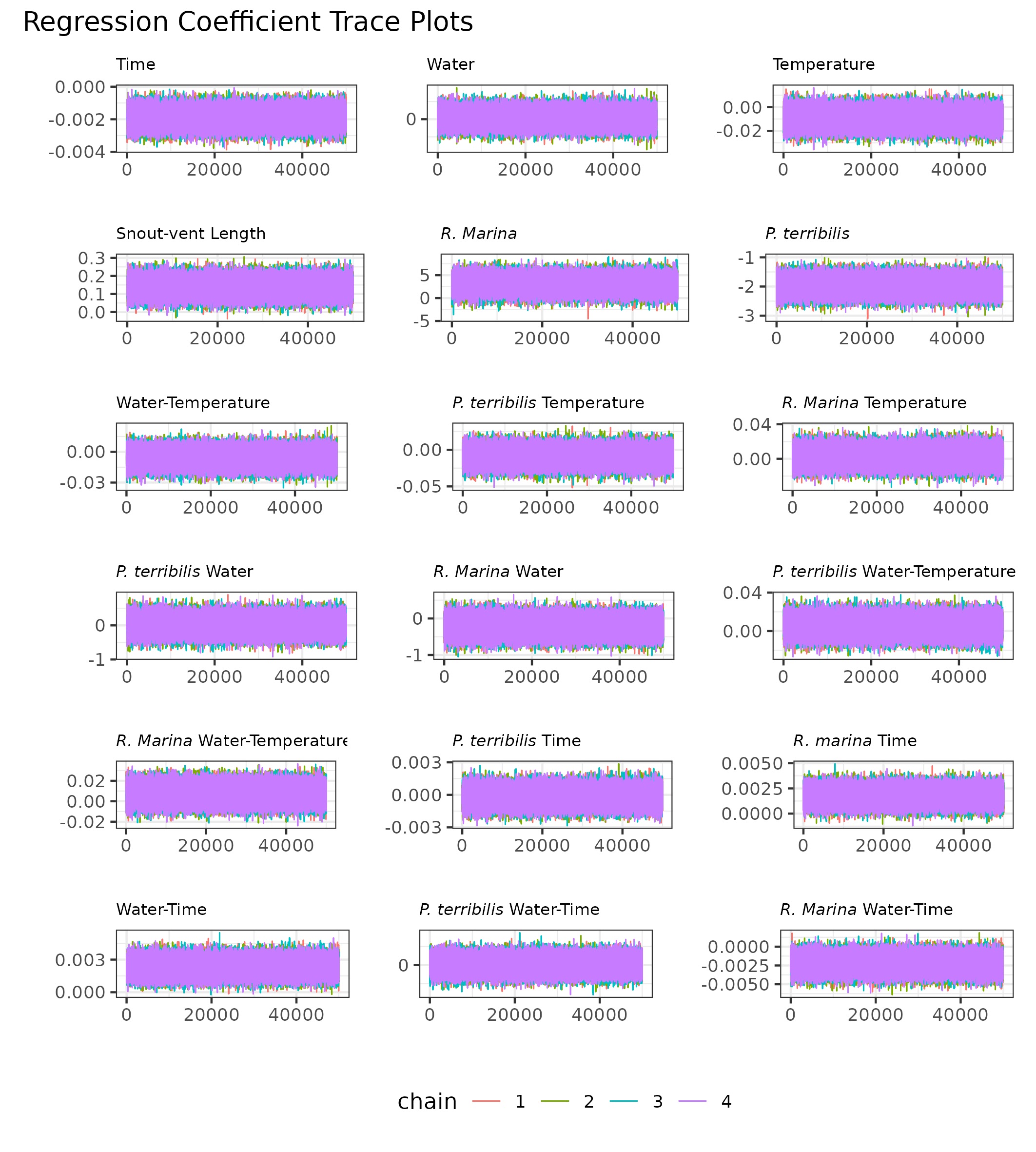
**Figure S3. Trace plot of the regression coefficients.** The y-axis corresponds to sampled coefficeint values across each model generation labeled on the x-axis. We used 50,000 generations in the model. The 4 colors in the plots correspond to each of the 4 chains in the model. Each chain shows no trend and similar mixing properties.

**Alt text:** 18 plots corresponding to the trace plots of each regression coefficient. The sampled values are on the y-axis and the model generation is on the x-axis of each plot. The data for each coefficient and chain is similar with no trend across generations.


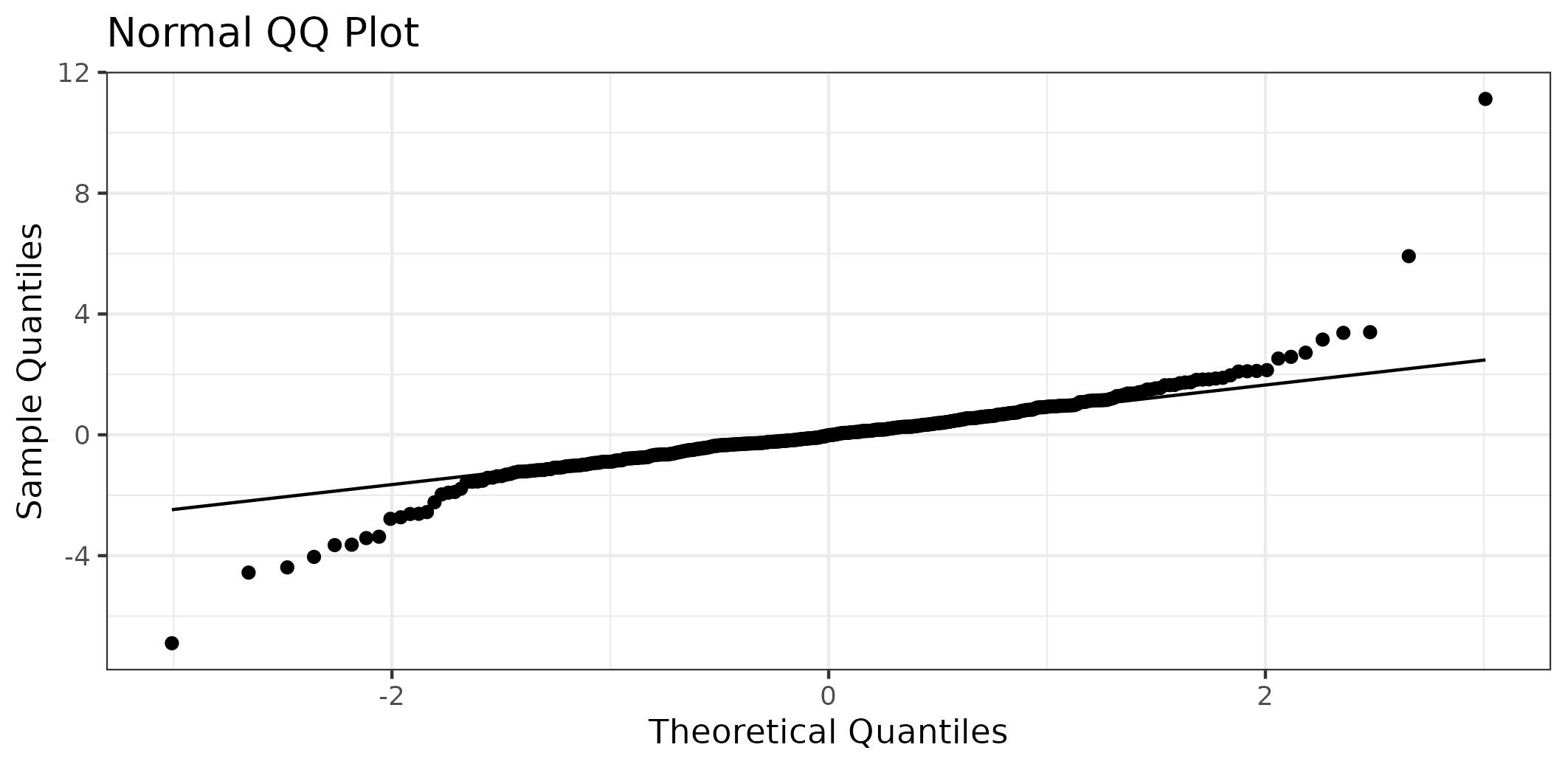


**Figure S4. QQ plot for model residuals.** Each point is a residual and the solid line is the theoretical 1-1 line.

**Alt text:** The y-axis shows sample quantiles and the x-axis shows the theoretical quantiles. The residuals perfectly match the theoretical 1-1 line in the middle of the plot, but show some deviations from the 1-1 line at lower and higher quantiles.


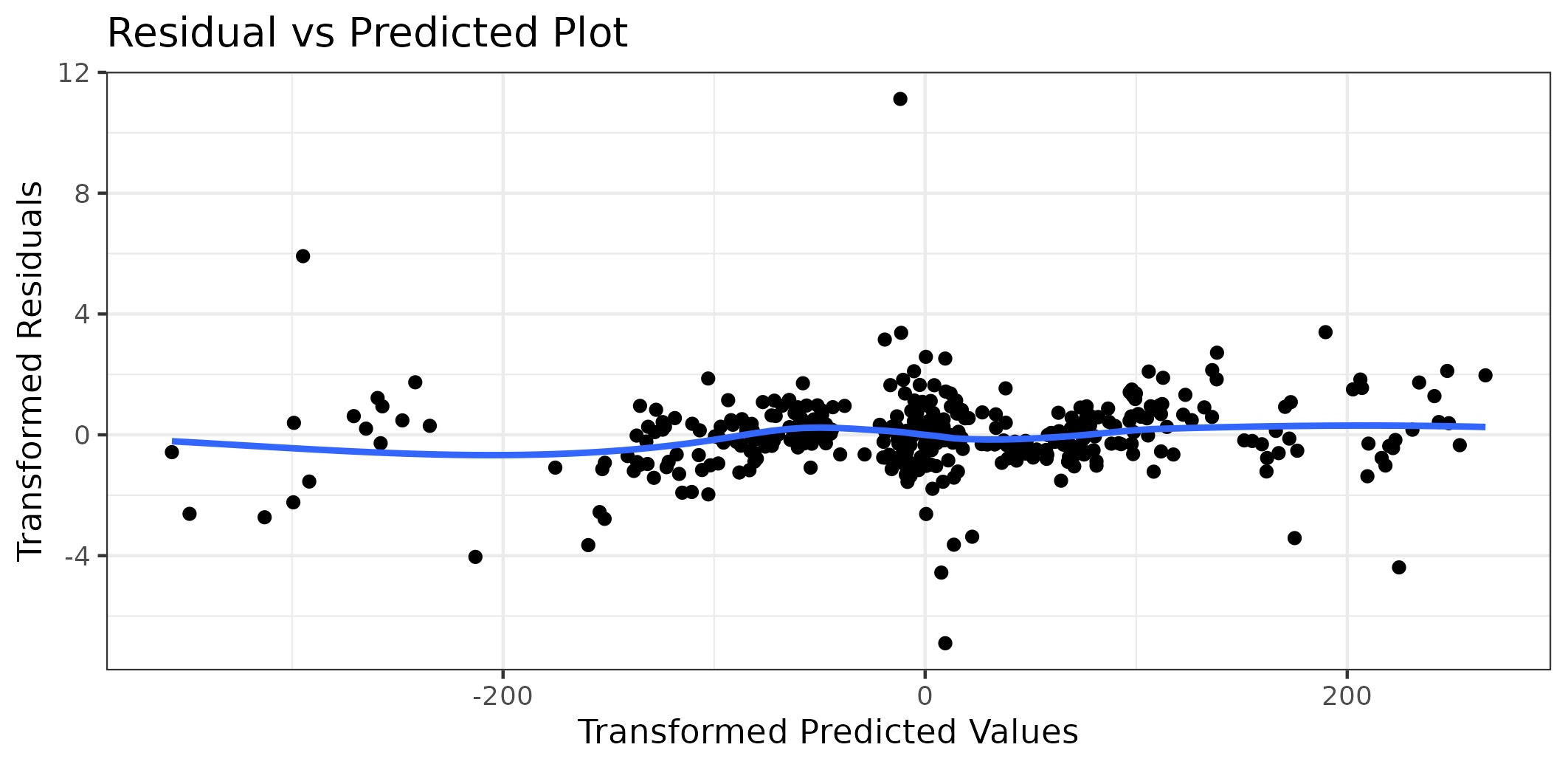


**Figure S5. Residual vs predicted plot for model residuals.** Each point is a residual and the blue line is the best fit smoothed line through the data.

**Alt text:** The y-axis is the transformed residuals and the x-axis is the transformed predicted values. The best fit smoothed line is flat at 0 with minor deviations throughout the range of the predicted values. The plot shows no trend.


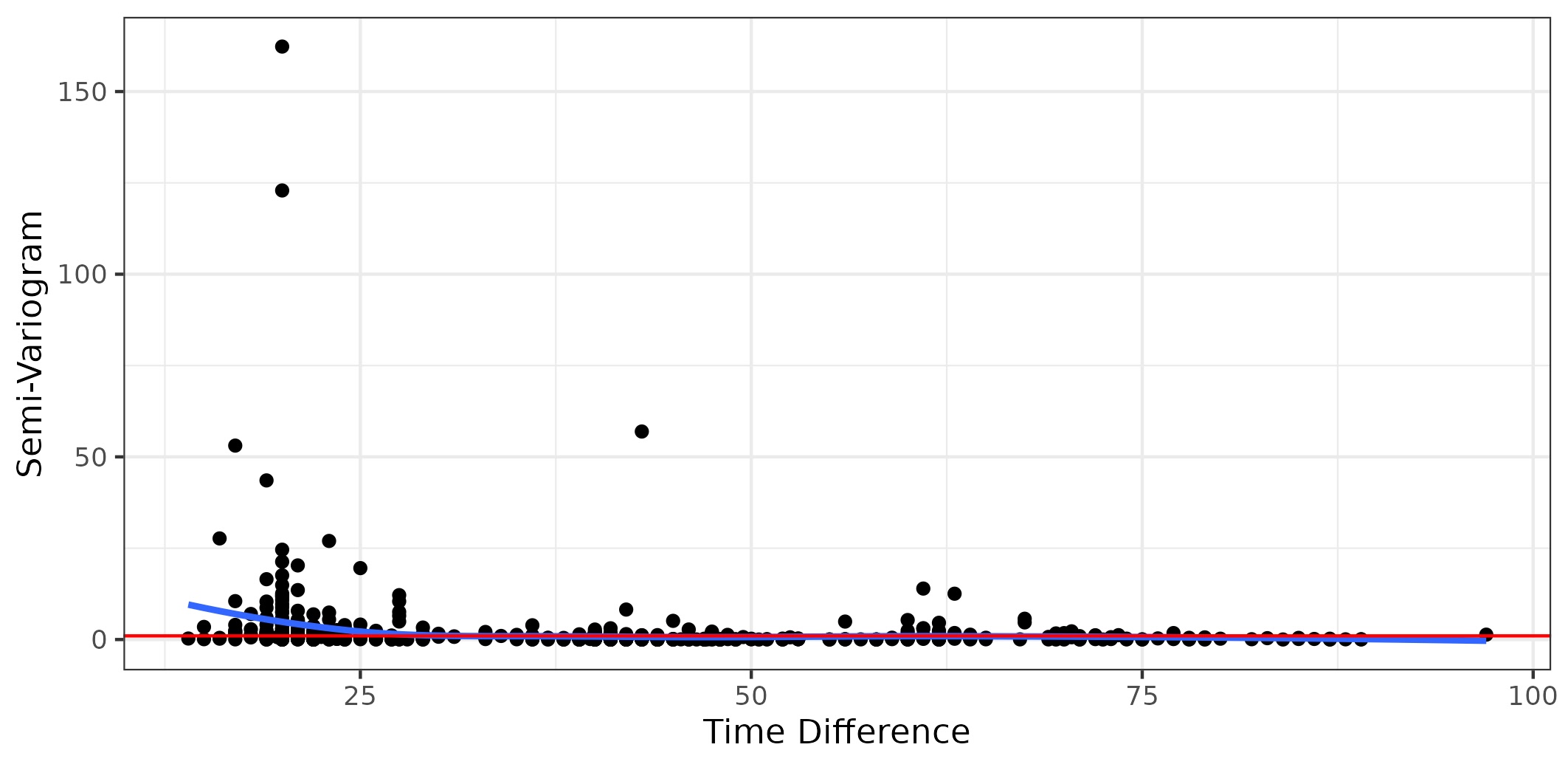


**Figure S6. Semi-variogram showing autocorrelation of sampled points.** The blue line is the best fit smoothed line for our data. The red (flat) line corresponds to an autocorrelation of 0.

**Alt text:** Semi-variogram values are on the y-axis and time differences are on the x-axis. The best fit line closely matches the line depicting no autocorrelation with only a small deviation at low time differences.
